# Neuroimmunomodulation in chronic wounds: an opinion

**DOI:** 10.3389/fcell.2025.1562346

**Published:** 2025-03-28

**Authors:** Patrizia Nardini, Stefano Bacci

**Affiliations:** ^1^ Research Unit of Histology and Embriology, Department of Clinical and Experimental Medicine, University of Florence, Florence, Italy; ^2^ Research Unit of Histology and Embriology, Department of Biology, University of Florence, Florence, Italy

**Keywords:** acute wounds, cellular infiltrate, chronic wounds, wound healing, neuromodulation

## 1 Introduction

The skin’s integrity and functionality are repaired through a series of processes that are conducted in a systematic and timely manner during wound healing (WH). Hemostasis, inflammation, proliferation, and remodeling are the four phases of WH that overlap (Fernández-Guarino et al., 2023a). The process of wound regeneration following an injury is significantly influenced by neurogenic stimuli; this is illustrated by the discovery that delayed wound repair occurs in animal models after the surgical removal of cutaneous nerves (Cheret et al., 2013; Laverdet et al., 2015; Martin and Nunan, 2015; Ashrafi et al., 2016; Kiya and Kubo, 2019; Oleszycka et al., 2022; Fernández-Guarino et al., 2023b). In addition, recent studies suggest that glial support cells may be key players in wound repair, and skin wounding triggers glial dedifferentiation and proliferation. In confirmation of this hypothesis, ablation of injury-activated glia leads to reduced tumor growth factor (TGF) β signaling and impaired wound repair (Peña and Martin, 2024). Studies in mice show that cutaneous nerve endings and Schwann cells surround a population of Lgr6+ epidermal stem cells in hair follicles, and denervation leads to the shifting of these cells toward a differentiated state, impairing wound re-epithelialization. Sensory nerves in the skin are also a source of Sonic Hedgehog (Shh), which induces Gli1 expression in cells of the hair follicle that contribute to WH by becoming epidermal stem cells (Peña and Martin, 2024). Skin wounding may damage the cutaneous vasculature and innervation, with axotomy studies in zebrafish enabling live imaging of the clearance of axon debris by macrophages and epidermal cells. The relationship between innervation and repair is reciprocal, with wounding triggering increased cutaneous nerve sprouting in some contexts (Peña and Martin, 2024).

## 2 Neuromodulation of wound healing

### 2.1 Phase of inflammation

The activation of critical processes during this phase of WH has been demonstrated by the release of numerous neuropeptides from cutaneous innervation. Substance P (SP) appears to be a significant factor in the inflammatory phase; however, other neuropeptides have also been identified and are reviewed. SP induces microvascular permeability and vasodilation by increasing the release of nitric oxide and exerting direct effects on endothelial cells. The expression of adhesion molecules on endothelial cells, monocyte chemotaxis, and inflammatory cell activity is upregulated by SP. SP also regulates the synthesis and release of pro-inflammatory cytokines, including interleukins, TGFα, and tumor necrosis factor (TNF)α, which are essential components during the inflammatory phase of wound healing. Neutral endopeptidase (NEP) is a zinc metalloprotease that inhibits the actions of SP through enzymatic degradation and competes with neurokinin-1 receptor 1(NK-1R). Inflammatory signaling in wound repair is significantly influenced by the interactions between SP and NEP (Roosterman et al., 2006; Cheret et al., 2013; Laverdet et al., 2015; Martin and Nunan, 2015; Ashrafi et al., 2016; Kiya and Kubo, 2019; Oleszycka et al., 2022; Fernández-Guarino et al., 2023a; Ivanov et al., 2023; Noble et al., 2024).

Neurokinin A (NKA) is a bioactive tachykinin released into the skin following an injury. It activates cutaneous target cells, including keratinocytes and dermal endothelial cells, preferentially through the neurokinin-2 receptor (NK-2R), thereby contributing to the regulation of skin inflammation during WH (Roosterman et al., 2006; Cheret et al., 2013; Laverdet et al., 2015; Martin and Nunan, 2015; Ashrafi et al., 2016; Kiya and Kubo, 2019; Oleszycka et al., 2022; Fernández-Guarino et al., 2023b; Ivanov et al., 2023; Noble et al., 2024).

Sensory cutaneous nerves contain corticotropin-releasing hormone (CRH), as evidenced by immunohistochemistry investigations. CRH induces skin mast cell (MC) degranulation and operates as a pro-inflammatory mediator, thereby increasing vascular permeability and the release of pro-inflammatory cytokines. CRH has also been demonstrated to promote angiogenesis in the epidermis (Roosterman et al., 2006; Cheret et al., 2013; Laverdet et al., 2015; Martin and Nunan, 2015; Ashrafi et al., 2016; Kiya and Kubo, 2019; Oleszycka et al., 2022; Fernández-Guarino et al., 2023a; Ivanov et al., 2023; Noble et al., 2024).

Calcitonin gene-related peptide (CGRP) is a vasodilator that can stimulate angiogenesis and improve plasma extravasation. CGRP has been demonstrated to enhance the inflammatory response of other mediators, including SP. Activin, a member of the TGF-β superfamily, has been demonstrated to upregulate CGRP expression in innervating sensory neurons and to increase after wounding, underscoring its regulatory function in WH (Roosterman et al., 2006; Cheret et al., 2013; Laverdet et al., 2015; Martin and Nunan, 2015; Ashrafi et al., 2016; Kiya and Kubo, 2019; Oleszycka et al., 2022; Fernández-Guarino et al., 2023b; Ivanov et al., 2023; Noble et al., 2024).

The role of nerve growth factor (NGF) as a modulator of the inflammatory phase of WH is underscored by its ability to increase the release of CGRP from peripheral nerve terminals into peripheral tissue (Roosterman et al., 2006; Cheret et al., 2013; Laverdet et al., 2015; Martin and Nunan, 2015; Ashrafi et al., 2016; Kiya and Kubo, 2019; Oleszycka et al., 2022; Fernández-Guarino et al., 2023a; Ivanov et al., 2023; Noble et al., 2024).

The function of neuropeptide Y (NPY) and CGRP in WH has been demonstrated in mouse models and is associated with its pro- and anti-inflammatory features. This role involves macrophage-derived NPY within the adipose tissue, a powerful modulator of inflammation, and fosters chemotaxis and angiogenesis (Roosterman et al., 2006; Cheret et al., 2013; Laverdet et al., 2015; Martin and Nunan, 2015; Ashrafi et al., 2016; Kiya and Kubo, 2019; Oleszycka et al., 2022; Fernández-Guarino et al., 2023b; Ivanov et al., 2023; Noble et al., 2024).

**FIGURE 1:**
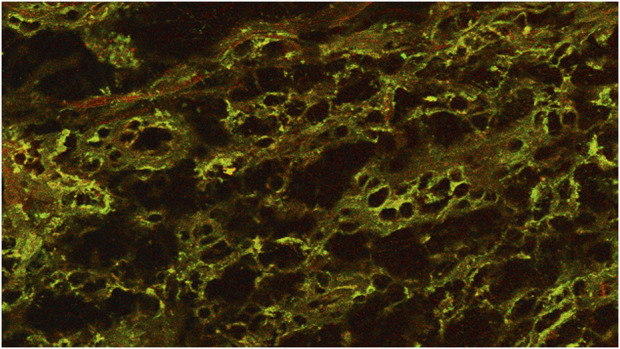
Coexpression of iNos in enolase-labeled unipolar neurons in a chronic wound. Confocal microscope 20×.

In recent years, the list of mediators involved in the process of WH has been expanded to include nitric oxide (NO), an extracellular molecular messenger. Nitric oxide synthase (NOs), an enzyme complex, is responsible for the production of NO, which is distinguished by an overregulation of the inducible isoform in response to stress. In reality, the enzyme’s production is elevated in the presence of inflammatory mediators, apoptotic corpses, or bacterial antigens. Consequently, it has been postulated that iNOs is involved in the inflammatory phase of wound repair, a phase during which it increases vasodilation and antibacterial activity. As a result, it has been demonstrated that iNOS plays a function in the process of wound repair (Nardini et al., 2024) (Figure 1) (See Table 1).

**TABLE 1:** Different phases of wound healing and mediators involved.

Phase of wound healing	Mediators	References
Inflammation	SP NKA CRH CGRP NGF NPY NO	Roosterman et al. (2006), Cheret et al. (2013), Laverdet et al. (2015), Martin and Nunan (2015), Ashrafi et al. (2016), Kiya and Kubo (2019), Oleszycka et al. (2022), Fernández-Guarino et al. (2023b), Ivanov et al. (2023), Noble et al. (2024), Nardini et al. (2024)
Proliferation	SP NGF GRP CGRP Galanin VIP PACAP	Roosterman et al. (2006), Cheret et al. (2013), Laverdet et al. (2015), Martin and Nunan (2015), Ashrafi et al. (2016), Kiya and Kubo (2019), Oleszycka et al. (2022), Fernández-Guarino et al. (2023a), Ivanov et al. (2023), Noble et al. (2024)
Remodeling	SP NGF NT3 BDNF CGRP VIP	Roosterman et al. (2006), Cheret et al. (2013), Laverdet et al. (2015), Martin and Nunan (2015), Ashrafi et al. (2016), Kiya and Kubo (2019), Oleszycka et al. (2022), Fernández-Guarino et al. (2023b), Ivanov et al. (2023), Noble et al. (2024)

### 2.2 Phase of proliferation

SP stimulates DNA synthesis, which results in potent proliferative effects on fibroblasts, keratinocytes, and endothelial cells. It also promotes angiogenesis through the potential mediation of NO. SP is essential for the remodeling of granulation tissue by promoting the proliferation and migration of dermal fibroblasts and by enhancing the expression of epidermal growth factor and its associated receptor (Roosterman et al., 2006; Cheret et al., 2013; Laverdet et al., 2015; Martin and Nunan, 2015; Ashrafi et al., 2016; Kiya and Kubo, 2019; Oleszycka et al., 2022; Fernández-Guarino et al., 2023a; Ivanov et al., 2023; Noble et al., 2024).

Polypeptide neurotrophins, such as NGF, are present in the central and peripheral nervous systems, as well as in a variety of cells, such as fibroblasts, epithelial cells, keratinocytes, and immune cells. NGF plays a critical role in the survival, function, and differentiation of sensory and autonomic nerves. Additionally, NGF exhibits anti-inflammatory properties. In particular, NGF has been hypothesized to facilitate the proliferation of local immature cells in lesions, the formation of blood vessels, and neurite overgrowth. In animal studies and a human case study, NGF has been demonstrated to facilitate epithelial healing and angiogenesis. The secretion of NGF in the epidermis is induced by neurokinin A (Roosterman et al., 2006; Cheret et al., 2013; Laverdet et al., 2015; Martin and Nunan, 2015; Ashrafi et al., 2016; Kiya and Kubo, 2019; Oleszycka et al., 2022; Fernández-Guarino et al., 2023b; Ivanov et al., 2023; Noble et al., 2024).

Additional neuropeptides that have been identified as contributing to the proliferative phase include gastrin-releasing peptide (GRP), CGRP, galanin, vasoactive intestinal peptide (VIP), and pituitary adenylate cyclase-activating peptide (PACAP).

CGRP is extensively distributed throughout the central and peripheral nervous system. While its potential to facilitate WH is uncertain, certain studies have demonstrated that it may facilitate angiogenesis, proliferation of keratinocytes, and migration. Keratinocyte proliferation and migration are facilitated by CGRP (Roosterman et al., 2006; Cheret et al., 2013; Laverdet et al., 2015; Martin and Nunan, 2015; Ashrafi et al., 2016; Kiya and Kubo, 2019; Oleszycka et al., 2022; Fernández-Guarino et al., 2023a; Ivanov et al., 2023; Noble et al., 2024).

Galanin is a peptide that is released from afferent nerves and has anti-proliferative effects in tissue. It signals through G-protein coupled receptors. In contrast, an *in vitro* study demonstrated that galanin stimulated the upregulation of NGF (Roosterman et al., 2006; Cheret et al., 2013; Laverdet et al., 2015; Martin and Nunan, 2015; Ashrafi et al., 2016; Kiya and Kubo, 2019; Oleszycka et al., 2022; Fernández-Guarino et al., 2023b; Ivanov et al., 2023; Noble et al., 2024).

Vasoactive intestinal peptide (VIP) has been demonstrated to function as a growth factor for the proliferation of keratinocytes and as a modulator of their migration. Additionally, VIP induces the release of histamine by MC, which results in vasodilation. VIP may be involved in the re-innervation of traumatized tissue as it has been demonstrated to accelerate the regeneration of the sciatic nerve in rats following transection. SP, CGRP, and VIP, as demonstrated by Cheret et al. (2013), modulate matrix metalloproteinase (MMP) activities and influence collagen-1 and collagen-3 production during cutaneous WH (Roosterman et al., 2006; Cheret et al., 2013; Laverdet et al., 2015; Martin and Nunan, 2015; Ashrafi et al., 2016; Kiya and Kubo, 2019; Oleszycka et al., 2022; Fernández-Guarino et al., 2023a; Ivanov et al., 2023; Noble et al., 2024).

PACAP is located in sensory cutaneous nerves. It is a potent vasodilator and a member of the VIP peptide family. In response to neuronal activation, it is hypothesized that C-fibers release PACAP, which in turn results in extravasation and vasodilation. PACAP is involved in cutaneous inflammation by promoting the proliferation of human keratinocytes and producing histamine from MC (Roosterman et al., 2006; Cheret et al., 2013; Laverdet et al., 2015; Martin and Nunan, 2015; Ashrafi et al., 2016; Kiya and Kubo, 2019; Oleszycka et al., 2022; Fernández-Guarino et al., 2023b; Ivanov et al., 2023; Noble et al., 2024) (See Table 1).

### 2.3 Remodeling phase

The function of neuropeptides or cutaneous innervation during the remodeling phase is poorly understood. In response to neuropeptides, sensory nerve fibers regenerate within the epidermis and dermis that have been repaired (Roosterman et al., 2006; Cheret et al., 2013; Laverdet et al., 2015; Martin and Nunan, 2015; Ashrafi et al., 2016; Kiya and Kubo, 2019; Oleszycka et al., 2022; Fernández-Guarino et al., 2023a; Ivanov et al., 2023; Noble et al., 2024).

SP induces the production of NGF by human dermal microvascular endothelial cells *in vitro*, a process that is necessary for the regeneration of nerve fibers following cutaneous injury. NGF has also been proposed to expedite tissue remodeling. Sensory and sympathetic nerves express neurotrophin-3 (NT3), a neurotrophic growth factor that is crucial for the growth, proliferation, and maintenance of nerves (Roosterman et al., 2006; Cheret et al., 2013; Laverdet et al., 2015; Martin and Nunan, 2015; Ashrafi et al., 2016; Kiya and Kubo, 2019; Oleszycka et al., 2022; Fernández-Guarino et al., 2023b; Ivanov et al., 2023; Noble et al., 2024).

Similarly, the postnatal survival or functional maturation of sensory neurons necessitates brain-derived neurotrophic factor (BDNF). Keratinocytes, fibroblasts, and myofibroblasts express BDNF and its receptors, which facilitate their proliferation and differentiation (Roosterman et al., 2006; Cheret et al., 2013; Laverdet et al., 2015; Martin and Nunan, 2015; Ashrafi et al., 2016; Kiya and Kubo, 2019; Oleszycka et al., 2022; Fernández-Guarino et al., 2023a; Ivanov et al., 2023; Noble et al., 2024).

SP has been demonstrated to affect the degradation of wound collagen by increasing the activity of MMP-2 in fibroblasts. As demonstrated by Fujiwara et al. *in vitro*, direct neuronal contact accelerates the differentiation of fibroblasts into myofibroblasts, which subsequently secrete collagen fibers and elicit wound contraction. In addition, MMP activities and collagen-1 and collagen-3 productions are influenced by SP, CGRP, and VIP during cutaneous WH (Roosterman et al., 2006; Cheret et al., 2013; Laverdet et al., 2015; Martin and Nunan, 2015; Ashrafi et al., 2016; Kiya and Kubo, 2019; Oleszycka et al., 2022; Fernández-Guarino et al., 2023b; Ivanov et al., 2023; Noble et al., 2024) (See Table 1).

## 3 Neuromodulation in chronic wounds

Chronic cutaneous lesions persist for 6 weeks to 8 weeks and exhibit an inflammatory response that maintains a balance between productive and degenerative processes. However, they do not adhere to the conventional, orderly, and timely repair process and progress through these phases without regaining the anatomical and functional integrity of the tissue. The process is impeded by numerous factors, with 140 pathologies implicated. Approximately 6.85% of individuals over 65 have at least one chronic illness, and 30% have three or more chronic conditions (Falanga et al., 2022; Fernández-Guarino et al., 2023a).

Skin ulcers are caused by various clinical symptoms and syndromes, but this investigation does not address the treatment of these conditions. Protease activity increases during the protracted inflammatory phase of ulcers, resulting in the disintegration of growth factors and other molecular signals that facilitate the reparative phase. The overproduction of pro-inflammatory cytokines and hydrolytic enzymes in chronic ulcers impedes the dominance of reparative processes over destructive ones (Martin and Nunan, 2015; Raziyeva et al., 2021; Grandi et al., 2022).

The body must maintain a balance between the synthesis of new tissue and the breakdown of old tissue in order to recuperate. Fibrinolytic systems and matrix metalloproteinases (MMPs) collaborate to eliminate fibrin and damaged extracellular matrix (ECM) from acute lesions, but lower tissue inhibitors of metalloproteinase (TIMP) concentrations and elevated MMP levels have been observed in chronic cutaneous lesions. This results in the matrix undergoing a subsequent reorganization, which exacerbates its degradation (Martin and Nunan, 2015; Raziyeva et al., 2021; Grandi et al., 2022).

Chronic WH mechanisms are similar to acute WH, but the dysregulation of MMP production is significantly associated with chronic wounds (CW), prolonging the inflammatory phase. Neutrophils are dispersed throughout the wound and emit a significant quantity of MMP, impairing the connective tissue matrix and elastase and deactivating vital proteins essential to the healing process (Martin and Nunan, 2015; Raziyeva et al., 2021; Grandi et al., 2022).

The immune system’s interactions with the nervous system are noteworthy in their ability to regulate WH processes (Chiu et al., 2012; Grandi et al., 2022). Recent research indicates that the transmission of neurotransmitters such as SP, protein gene product 9.5 (PGP 9.5), NO, NGF, NKA, NPY, CGRP, and VIP are crucial in chronic wounds, and such transmission is facilitated by interactions between MC and nerve cells, resulting in the release of ECM by fibroblasts, the elevation of TGFβ levels, and the reaction of infiltrating cells (Grandi et al., 2021).

Infections are a significant impediment to the healing process, as they prolong the inflammatory phase and lead to elevated MMP levels. Bacteria form polysaccharide biofilms, which are shielded from antibiotics by reduced penetration through the biofilm matrix, and genetic mutations modify their susceptibility to antibiotics. Biofilms periodically release single bacterial cells that can colonize new surfaces or degrade the collagen matrix in healed ulcers, a process known as “re-ulceration.” The cells that remain in the colony, die, or depart can be influenced by variations in the conditions within the biofilm.

Clinical lesions are generally more subdued when infected due to the release of endotoxins and proteases, disrupting the extracellular matrix and releasing mediators that exacerbate the local inflammatory response. This is a potential correlation between infection and an increase in exudate, which is inhibited by elevated levels of macromolecules, such as albumin and fibrin, as well as pro-inflammatory cytokines and MMP.

Photodynamic treatment (PDT) therapy for CW has been shown to increase the density of neuronal populations in the dermis, which are a component of the autonomous nervous system and contain the typical nerve mediators already defined for chronic wounds. The proportion of mast cells capable of secreting and containing NGF and VIP and iNOs compounds increases as a result of a single irradiation, consistent with the previously identified increase in mast cell degranulation index (Grandi et al., 2021; Grandi et al., 2022; Nardini et al., 2024).

## 4 Conclusion

Neuromodulation in WH is a concept of extreme interest. In fact, it is important to recognize that all the phases that characterize this important process have not yet been fully clarified, and it would not be surprising if, in the future, other mediators could be discovered. On the other hand, WH is itself a complex process, and it is no coincidence that the term neuromodulation is often replaced by the term neuroimmunomodulation, underlining how the cells of the immune system are involved in the various phases. An example is given by MCs, which are mainly located near nerves and play a role in WH. When an article proposed how the release of histamine stimulated that of acetylcholine, numerous publications followed the idea (Fantozzi et al., 1978).

The concept of neuroimmunomodulation becomes more complicated in chronic wounds treated with photodynamic therapy because, in this case, the nervous mediators are able to stimulate the secretion of the extracellular matrix by fibroblasts and that of the cellular infiltrate. It is precisely in this location that the mast cell expresses NGF, and this means that the increased content of this mediator in these cell types is fundamental for the possible healing of the wound (Siiskonen and Harvima, 2019; Grandi et al., 2021; Nardini et al., 2024). We are, therefore, faced with an incredible flow of ideas that must be placed in the right relationships to begin to understand the complexity of this phenomenon.

## 5 Prospective research

Advanced imaging techniques and high-throughput screening could be beneficial in studying neuroimmunomodulation in chronic wound healing.

An investigation into the molecular interactions that occur between neuropeptides, immune cells, and signaling pathways may allow for the discovery of new treatment options.

Additional clinical trials are required to determine whether PDT is effective in promoting wound healing through neuroimmunomodulation, its efficacy, and its probable mechanisms. This might provide a more detailed roadmap for further research studies and medicinal uses.
